# Risk of thoracic soft tissue sarcoma after breast cancer radiotherapy: a population-based cohort study in Osaka, Japan

**DOI:** 10.1093/jrr/rrae010

**Published:** 2024-03-21

**Authors:** Toshiki Ikawa, Yoshihiro Kuwabara, Kayo Nakata, Naoyuki Kanayama, Masahiro Morimoto, Isao Miyashiro, Koji Konishi

**Affiliations:** Department of Radiation Oncology, Osaka International Cancer Institute, 3-1-69 Otemae, Chuo-ku, Osaka 541-8567, Japan; Cancer Control Center, Osaka International Cancer Institute, 3-1-69 Otemae, Chuo-ku, Osaka 541-8567, Japan; Cancer Control Center, Osaka International Cancer Institute, 3-1-69 Otemae, Chuo-ku, Osaka 541-8567, Japan; Department of Radiation Oncology, Osaka International Cancer Institute, 3-1-69 Otemae, Chuo-ku, Osaka 541-8567, Japan; Department of Radiation Oncology, Osaka International Cancer Institute, 3-1-69 Otemae, Chuo-ku, Osaka 541-8567, Japan; Cancer Control Center, Osaka International Cancer Institute, 3-1-69 Otemae, Chuo-ku, Osaka 541-8567, Japan; Department of Radiation Oncology, Osaka International Cancer Institute, 3-1-69 Otemae, Chuo-ku, Osaka 541-8567, Japan

**Keywords:** treatment-related neoplasms, breast neoplasm, radiotherapy, sarcoma, neoplasms, second primary

## Abstract

Postoperative radiotherapy for breast cancer reportedly increases the risk of thoracic soft tissue sarcomas, particularly angiosarcomas; however, the risk in the Japanese population remains unknown. Therefore, this study aimed to investigate the incidence of thoracic soft tissue sarcoma among patients with breast cancer in Japan and determine its association with radiotherapy. This retrospective cohort study used data from the population-based cancer registry of the Osaka Prefecture. The inclusion criteria were female sex, age 20–84 years, diagnosis of breast cancer between 1990 and 2010, no supraclavicular lymph node or distant metastasis, underwent surgery and survived for at least 1 year. The primary outcome was the occurrence of thoracic soft tissue sarcomas 1 year or later after breast cancer diagnosis. Among the 13 762 patients who received radiotherapy, 15 developed thoracic soft tissue sarcomas (nine angiosarcomas and six other sarcomas), with a median time of 7.7 years (interquartile range, 4.0–8.6 years) after breast cancer diagnosis. Among the 27 658 patients who did not receive radiotherapy, four developed thoracic soft tissue sarcomas (three angiosarcomas and one other sarcoma), with a median time of 11.6 years after diagnosis. The 10-year cumulative incidence was higher in the radiotherapy cohort than in the non-radiotherapy cohort (0.087 *vs.* 0.0036%, *P* < 0.001). Poisson regression analysis revealed that radiotherapy increased the risk of thoracic soft tissue sarcoma (relative risk, 6.8; 95% confidence interval, 2.4–24.4). Thus, although rare, breast cancer radiotherapy is associated with an increased risk of thoracic soft tissue sarcoma in the Japanese population.

## INTRODUCTION

Breast cancer is the most prevalent cancer among women worldwide, including in Japan, with 97 142 cases recorded in 2019 [1]. The current standard treatment for early stage breast cancer is conservative surgery, followed by postoperative radiotherapy. A meta-analysis has shown that postoperative radiotherapy reduces breast cancer recurrence and improves breast cancer-specific survival [2]. Moreover, in patients with lymph node metastases, postoperative irradiation of the regional lymph node area in addition to that of the breast or chest wall reduces recurrence and improves breast cancer-specific or overall survival [3, 4]. Thus, radiotherapy plays a key role in the treatment of breast cancer, with the current 10-year relative survival rate for localized breast cancer exceeding 90% [5].

However, radiation-associated sarcoma is a rare but life-threatening secondary cancer that affects breast cancer survivors [6, 7]. Thus, there is an increasing need to carefully consider radiotherapy-related secondary cancers as potential late adverse events. Population-based studies show that postoperative radiotherapy increases the risk of sarcomas, particularly angiosarcomas, in breast cancer survivors, although their incidence is notably low [8–10]. However, these studies originated predominantly in the USA and Europe. Given the potential influence of lifestyle and genetic background variations on secondary cancer development, an investigation of radiotherapy-associated sarcoma among patients with breast cancer in Japan is crucial. Therefore, this study aimed to investigate the association between the incidence of thoracic soft tissue sarcoma (STS) and radiotherapy among patients with breast cancer in Japan, using data from a population-based cancer registry.

## MATERIALS AND METHODS

### Study design and patients

This retrospective cohort study was approved by the ethics committee of Osaka International Cancer Institute (approval number, 23003–2).

We extracted breast cancer cases diagnosed between 1990 and 2010 from the Osaka Cancer Registry. Briefly, the Osaka Cancer Registry is a population-based cancer registry that targets all cancers in the Osaka Prefecture, Japan. Established in 1962, it has a 60-year history as one of Japan’s longest-running databases. Moreover, it meets the international criteria for comparability, completeness, and validity [11] and encompasses the entire population of Osaka Prefecture, Japan’s third most populous region (with 8.8 million residents as of 2015) [12]. Follow-up surveys of survival information are conducted using records from medical facilities, death certificate databases, and the inhabitant’s registry.

The records include information about age; sex; survival information up to December 2019; date of death or last follow-up date of survival information; date of tumor diagnosis; progressive stage; the International Classification of Diseases, 10th Revision (ICD-10) codes; tumor site and histology codes according to the International Classification of Diseases for Oncology, third edition (ICD-O-3); and initial treatment types—surgery, radiotherapy, chemotherapy and hormone therapy—administered within 4 months of tumor diagnosis or already scheduled. Progressive stages of breast cancer are classified into five groups based on the extent of the disease: carcinoma *in situ*, localized, regional lymph node metastasis, adjacent organ invasion and distant metastasis. These stages correspond to the International Union Against Cancer Tumor–Node–Metastasis classification (seventh edition) for breast cancer as follows: TisN0M0, T1–3N0M0, T0–3N1–3bM0, T4N0–3bM0 and N3c or M1.

**Fig. 1:**
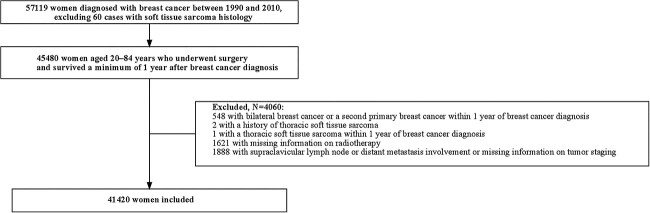
Patient selection flowchart.

In the database, the percentage of the breast cancer records (ICD-10 codes: D05.0–D05.9, and C50.0–C50.9) notified by death certificate-only (DCO) was 3.9% in 1990, 2.1% in 2000 and 2.8% in 2010. DCO records (2.6%) and those diagnosed at autopsy (0.0089%) were excluded from this study. Figure 1 illustrates the patient selection flowchart. In total, 57 119 women diagnosed with breast cancer were identified, excluding 60 cases with STS histology. The ICD-O-3 histology codes for STS were cited from the supplementary appendix of Veiga *et al.’s* study [8]. Among them, patients aged 20–84 years who underwent surgery and survived a minimum of 1 year after breast cancer diagnosis were eligible. The exclusion criteria were patients with bilateral breast cancer, a second primary breast cancer within 1 year of breast cancer diagnosis, or a history of prior thoracic STS or diagnosis within 1 year of breast cancer diagnosis. Additionally, patients who lacked information on radiotherapy or progressive stage, or had a progressive stage of distant metastasis were excluded.

### Outcomes

The primary outcome was the occurrence of thoracic STS 1 year or later after breast cancer diagnosis. The ICD-O-3 site codes for thoracic locations were cited from the supplementary appendix of Veiga *et al.’s* study [8]. The follow-up ended at the earliest of the following timepoints: initial thoracic STS diagnosis, diagnosis of second primary breast cancer (to negate the influence of treatment), age 100 years, last follow-up (31 December 2019) or death.

### Statistical analyses

First, differences in patient characteristics between the radiotherapy and non-radiotherapy cohorts were analyzed. The covariates included age at breast cancer diagnosis (20–49, 50–59 or 60–84 years), age at the end of follow-up (21–59, 60–69 or 70–100 years), calendar year of breast cancer diagnosis (1990–99 *vs* 2000–10), receipt of chemotherapy (no/unknown *vs* yes), receipt of hormone therapy (no/unknown *vs* yes) and progressive stage (early stage: carcinoma *in situ* or localized *vs* advanced stage: regional lymph node metastasis or adjacent organ invasion). Covariate balance was assessed using the Wilcoxon rank-sum test for continuous variables and the Fisher’s exact test or chi-squared test for categorical variables. Second, overall survival was measured using the Kaplan–Meier method starting 1 year after breast cancer diagnosis. The cumulative incidence of thoracic STS was estimated using the cumulative incidence function, accounting for death and second primary breast cancer as competing risks. Differences between the radiotherapy and non-radiotherapy cohorts were assessed using Gray’s test [13]. Third, multivariable Poisson regression analyses were performed to examine the association between radiotherapy and incidence of thoracic STS and to estimate the relative risk (RR). All analyses were performed using R software (version 4.2.3) (R Foundation for Statistical Computing, Vienna, Austria). All statistical tests were two-sided, and *P* < 0.05 was considered statistically significant.

## RESULTS

Overall, 41 420 patients were included in this study; among them, 13 762 and 27 658 underwent and did not undergo radiotherapy, respectively. Compared with the non-radiotherapy cohort, the radiotherapy cohort was younger at breast cancer diagnosis and at the end of follow-up, was treated in the modern era and received hormonal therapy more frequently, and presented with earlier stages of breast cancer (Table 1). The covariate proportions for radiotherapy and non-radiotherapy cohorts are detailed in Supplementary Table 1.

**Table 1:** Comparison of patient characteristics between the radiotherapy and the non-radiotherapy cohorts

	Overall	Radiotherapy		
Characteristic	*n* = 41 420^a^	Yes, *n* = 13 762^a^	No, *n* = 27 658^a^	*P*-value^b^
Median age at breast cancer diagnosis, years	56.0 (47.0, 66.0)	53.0 (46.0, 62.0)	57.0 (48.0, 67.0)	< 0.001
Age at breast cancer diagnosis, years				< 0.001
20–49	13 687 (33%)	5247 (38%)	8440 (31%)	
50–59	11 105 (27%)	3956 (29%)	7149 (26%)	
60–84	16 628 (40%)	4559 (33%)	12 069 (44%)	
Median age at the end of follow-up, years	70.0 (61.0, 79.0)	68.0 (59.0, 76.0)	71.0 (62.0, 81.0)	< 0.001
Age at the end of follow-up, years				< 0.001
21–59	9213 (22%)	3652 (27%)	5561 (20%)	
60–69	10 312 (25%)	3882 (28%)	6430 (23%)	
70–100	21 895 (53%)	6228 (45%)	15 667 (57%)	
Calendar year of breast cancer diagnosis				< 0.001
1990–99	14 288 (34%)	3974 (29%)	10 314 (37%)	
2000–10	27 132 (66%)	9788 (71%)	17 344 (63%)	
Chemotherapy				0.066
No/unknown	23 252 (56%)	7813 (57%)	15 439 (56%)	
Yes	18 168 (44%)	5949 (43%)	12 219 (44%)	
Hormone therapy				< 0.001
No/unknown	20 080 (48%)	5127 (37%)	14 953 (54%)	
Yes	21 340 (52%)	8635 (63%)	12 705 (46%)	
Progressive stage				< 0.001
Early	27 328 (66%)	9629 (70%)	17 699 (64%)	
Advanced	14 092 (34%)	4133 (30%)	9959 (36%)	
Median follow-up time since breast cancer diagnosis, years	12.8 (9.5, 18.9)	12.7 (9.9, 18.4)	12.9 (9.3, 19.3)	0.3
Follow-up time since breast cancer diagnosis, years				
1–4	4389 (11%)	1157 (8.4%)	3232 (12%)	
5–9	7691 (19%)	2463 (18%)	5228 (19%)	
10–14	13 192 (32%)	5022 (36%)	8170 (30%)	
15–19	7243 (17%)	2554 (19%)	4689 (17%)	
20–30	8905 (21%)	2566 (19%)	6339 (23%)	

^a^ *n* (%); median (IQR).

^b^Pearson’s Chi-squared test; Wilcoxon rank sum test.

**Fig. 2:**
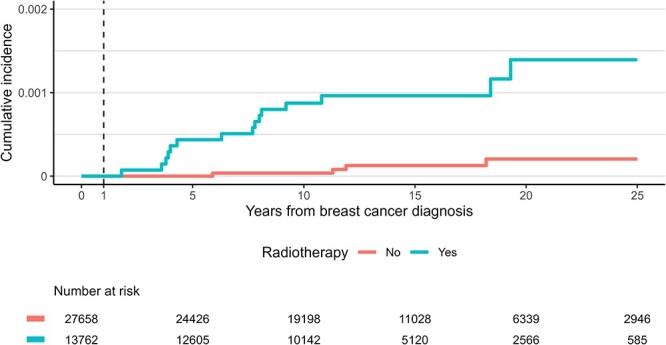
Cumulative incidence of thoracic STS.

The median follow-up time since breast cancer diagnosis was 12.7 years (interquartile range [IQR], 9.9–18.4 years) and 12.9 years (IQR, 9.3–19.3 years) in the radiotherapy and non-radiotherapy cohorts, respectively. The 10-year overall survival rate was 85.1 and 77.8% in the radiotherapy and non-radiotherapy cohorts, respectively. Thoracic STSs developed in 15 patients in the radiotherapy cohort with a median of 7.7 years (IQR, 4.0–8.6 years; range, 1.8–19.3 years) and in four patients in the non-radiotherapy cohort with a median time of 11.6 years (range, 5.9–18.2 years) (Tables 2 and 3). In the radiotherapy cohort, thoracic angiosarcomas developed in nine patients, and other subtypes of thoracic STS developed in six patients (Table 2). Meanwhile, three and one patients in the non-radiotherapy cohort had angiosarcoma and other subtypes, respectively (Table 3). The 10-year cumulative incidence of thoracic STS was 0.087% (95% confidence interval [CI], 0.048–0.15%) and 0.0036% (95% CI, 0.0004–0.021%) in the radiotherapy and non-radiotherapy cohorts, respectively (*P* < 0.001; Fig. 2). When divided between thoracic angiosarcoma and other subtypes, the 10-year cumulative incidence of thoracic angiosarcoma and other subtypes in the radiotherapy cohort was 0.051% (95% CI, 0.024–0.10%; Supplementary Fig. 1) and 0.036% (95% CI, 0.014–0.083%; Supplementary Fig. 2), respectively.

**Table 2:** Summary of thoracic STSs in patients who underwent radiotherapy for breast cancer

Characteristic	Overall, *N* = 15^a^	Angiosarcoma, *N* = 9^a^	Other subtypes, *N* = 6^a^^,^^b^
Site of thoracic STS^c^			
Breast	6 (40%)	4 (44%)	2 (33%)
Skin of trunk	3 (20%)	3 (33%)	0 (0%)
Soft tissues of thorax	3 (20%)	2 (22%)	1 (17%)
Lung	1 (6.7%)	0 (0%)	1 (17%)
Peripheral nerves and autonomic nervous system of trunk	1 (6.7%)	0 (0%)	1 (17%)
Rib, sternum, clavicle and associated joints	1 (6.7%)	0 (0%)	1 (17%)
Time to onset of thoracic STS, years^c^	7.7 [4.0, 8.6] (1.8–19.3)	7.8 [4.0, 9.2] (3.8–19.3)	7.0 [4.3, 7.9] (1.8–18.4)

^a^ *n* (%); median [IQR] (minimum–maximum).

^b^Other subtypes include carcinosarcoma, NOS; chondrosarcoma, NOS; liposarcoma, NOS; malignant fibrous histiocytoma; malignant peripheral nerve sheath tumor; and sarcoma, NOS.

^c^STS = soft tissue sarcoma.

**Table 3:** Summary of thoracic STSs in patients who did not undergo radiotherapy for breast cancer

Characteristic	Overall, *N* = 4^a^	Angiosarcoma, *N* = 3^a^	Other subtypes, *N* = 1^a^^,^^b^
Site of thoracic STS^c^			
Breast	1 (25%)	1 (33%)	0 (0%)
Skin of trunk	1 (25%)	1 (33%)	0 (0%)
Soft tissues of thorax	1 (25%)	1 (33%)	0 (0%)
Anterior mediastinum	1 (25%)	0 (0%)	1 (100%)
Time to onset of thoracic STS, years^c^	11.6 (5.9–18.2)	11.3 (5.9–11.9)	18.2

^a^ *n* (%); median (minimum–maximum).

^b^Other subtypes include small cell sarcoma.

^c^STS = soft tissue sarcoma.

Considering the impact of collinearity, the multivariable Poisson regression analysis included the age at the end of follow-up, rather than the age at breast cancer diagnosis. In the multivariable Poisson regression analyses, radiotherapy was significantly associated with thoracic STS occurrence (RR, 6.8; 95% CI, 2.4–24.4; Table 4). Similarly, radiotherapy demonstrated a significant association with the occurrence of thoracic angiosarcoma (RR, 6.7; 95% CI, 1.9–30.8; Supplementary Table 2) and other subtypes (RR, 10.4; 95% CI, 1.8–198; Supplementary Table 3). In the analyses that included age at breast cancer diagnosis instead of age at the end of follow-up, radiotherapy was also significantly associated with thoracic STS occurrence (Supplementary Table 4).

**Table 4:** Multivariable Poisson regression analysis of the RR of thoracic STSs

Characteristic	Person-years	Event N	RR^a^	95% CI^a^	*P*-value
Radiotherapy					
No	361 946	4	—	—	
Yes	175 768	15	6.83	2.41, 24.4	< 0.001
Age at the end of follow-up, years					
21–59	83 714	5	—	—	
60–69	133 101	4	0.61	0.15, 2.30	0.5
70–100	320 899	10	0.83	0.29, 2.70	0.7
Calendar year of breast cancer diagnosis					
1990–99	248 595	4	—	—	
2000–10	289 119	15	2.46	0.86, 8.90	0.12
Chemotherapy					
No/unknown	297 142	13	—	—	
Yes	240 573	6	0.65	0.21, 1.82	0.4
Hormone therapy					
No/unknown	246 012	7	—	—	
Yes	291 702	12	1.21	0.48, 3.31	0.7
Progressive stage					
Early	374 678	14	—	—	
Advanced	163 036	5	1.18	0.35, 3.43	0.8

^a^RR = relative risk, CI = confidence interval.

## DISCUSSION

This population registry-based study conducted in Japan found that postoperative patients with breast cancer undergoing radiotherapy had a significantly increased risk of developing thoracic STSs. Moreover, 9 of the 15 patients who developed thoracic STS after radiotherapy were diagnosed with angiosarcoma. Therefore, it is essential for physicians to be cognizant of the potential risk of thoracic STS development, particularly angiosarcomas, in patients undergoing radiotherapy, and to monitor these patients accordingly.

Thus, the risk of thoracic STS was increased after radiotherapy, consistent with previous findings reported in the USA and Europe [8–10]. Table 5 summarizes the current study and previous population-based studies on radiotherapy-associated STSs in patients diagnosed with breast cancer within similar time periods. The prevalence of thoracic STS after radiotherapy in patients diagnosed with breast cancer between 1990 and 2010 was low at 0.087% of patients over a 10-year period. Furthermore, the 10-year cumulative incidence of thoracic angiosarcoma after radiotherapy was 0.051%. Conversely, a US based study [8] of two cohort datasets from 1990–2016 and from 1992–2016 estimated the 10-year incidence of thoracic STSs at 0.21 and 0.15%, respectively, in patients undergoing radiotherapy for breast cancer. Moreover, a Dutch study [14] using cohort data from 1989–2015 reported that 1 in 1000 patients developed angiosarcoma after radiotherapy for breast cancer. Therefore, the impact of radiotherapy on the development of thoracic STS appears to be slightly lower in Japan compared with the USA and the Netherlands. This is because the crude incidence rate of STSs across all anatomical sites, including primary sarcomas, is equally rare in both Japan and Europe [15], suggesting that there is no clear difference in the spontaneous incidence rates of STSs between the two regions.

**Table 5:** Current and previous population-based studies on radiotherapy-associated STSs in patients diagnosed with breast cancer within similar time periods

Study (country)	Breast cancer diagnosis period	Number in radiotherapy cohort (overall cohort)	Median follow-up time for overall cohort, years (IQR)	Cumulative incidence in radiotherapy cohort (95% CI)	
				Thoracic STS	Thoracic angiosarcoma
This study (Japan)	1990–2010	13 762	12.8	0.087%^a^	0.051%^a^
		(41 420)	(9.5–18.9)	(0.048–0.15%)	(0.024–0.10%)
Veiga *et al.* [8] (USA)	1990–2016	10 638	9.3	0.21%^a^	0.14%^a^
		(15 940)	(5.7–13.9)	(0.12–0.34%)	(0.07–0.30%)
	1992–2016	253 286	8.3	0.15%^a^	0.11%^a^
		(457 300)	(4.3–13.9)	(0.13–0.17%)	(0.09–0.12%)
Rombouts *et al.* [14] (Netherlands)	1989–2015	184 823	7.7		0.1%
		(296 577)	(range, 0–28.1)		

^a^10-year cumulative incidence.

Angiosarcomas account for a considerable proportion of thoracic STS cases after breast radiotherapy [8, 10] and occur more frequently in patients who undergo breast-conserving surgery followed by postoperative radiotherapy than in those who undergo mastectomy plus radiotherapy [8, 14]. The potential onset of thoracic angiosarcoma has been suggested to be related to the worsening of postoperative breast edema due to radiotherapy [16, 17]. Around 1990, breast-conserving surgery accounted for 39% of breast cancer surgeries in one region of the USA and 36% of breast cancer patients in the Netherlands [18, 19]. In contrast, in Japan, breast-conserving surgery accounted for only 14.5% of breast cancer surgeries [20]. This disparity in the prevalence of breast-conserving surgery may potentially contribute to variations in the incidence of angiosarcoma. Nonetheless, the proportion of breast-conserving surgery among all breast cancer surgeries in Japan increased from 14.5% in 1992 to 40.8% in 2000 and 58.6% in 2011 [20, 21]. Thus, given the recent upsurge in breast-conserving surgery, future investigations to estimate the incidence of thoracic STSs using more recent long-term data are warranted.

Veiga *et al.* [8] reported that hypertension and diabetes, both linked to increased edema, along with breast-conserving surgery, are potential risk factors for the development of angiosarcoma. They also found that certain chemotherapy regimens, specifically anthracyclines and alkylating agents, are associated with an increased risk of angiosarcoma and other sarcomas, respectively. Notably, no significant association was found with radiotherapy dose, fractionation or boost use. However, our study lacked data on these potential factors. Therefore, further research is necessary to determine their impact on the development of angiosarcoma and other sarcomas in Japan.

This study had some limitations. First, this study only targeted rare malignancies, which may limit its generalizability. The number of patients who developed thoracic STSs was small, leading to a wide 95% CI of the RR in the Poisson regression analysis, although statistically significant. This suggests potential inaccuracies in estimating the RR. In addition, we did not include all covariates in the analysis focused on thoracic angiosarcoma and other subtypes of thoracic STS. The second limitation was incomplete data and lack of detailed information on treatment and patient background. The Osaka Cancer Registry categorizes radiotherapy as either administered within 4 months of tumor diagnosis or as already scheduled. Thus, patients who received radiotherapy beyond this 4-month window or who were not initially scheduled might have been inaccurately classified into the non-radiotherapy group. Therefore, the observed impact of radiotherapy on the development of thoracic STS may have been underestimated. Furthermore, cancer onset might have been missed for long-term survivors who relocated outside Osaka Prefecture or were documented as DCO cases. Moreover, detailed radiotherapy data were lacking, preventing the assessment of the effects of dose fractionation, irradiation area and irradiation method. The registry does not specify the timing of radiotherapy administration; thus, some patients may have undergone preoperative or intraoperative radiotherapy instead of postoperative radiotherapy. Therefore, our findings require further validation by establishing a population-based radiotherapy database linked to the cancer registry in Japan in the future. Finally, the strength of this study is that it examined the incidence of STSs in a cohort over a median of >10 years. The median latency period for the development of angiosarcoma associated with radiotherapy for breast cancer is <10 years [14, 16, 22]. Conversely, for radiation-associated sarcomas, it varies widely by histological type [23]. Thus, a longer follow-up is required to assess the impact of radiotherapy on the development of STSs other than angiosarcoma.

In conclusion, postoperative radiotherapy for breast cancer plays a crucial role in reducing breast cancer recurrence and improving patient prognosis. However, the risk of thoracic STSs is increased among patients with breast cancer who undergo radiotherapy although the incidence is low in Japan. This finding may provide better information for individual decision-making for patients with breast cancer in Japan.

## Supplementary Material

Supplemental_data_rrae010

## PRESENTATION AT A CONFERENCE

This study has not been presented at any conference.
