# Correction to: Risk of thoracic soft tissue sarcoma after breast cancer radiotherapy: a population-based cohort study in Osaka, Japan

**DOI:** 10.1093/jrr/rrae029

**Published:** 2024-05-08

This is a correction to: Toshiki Ikawa, Yoshihiro Kuwabara, Kayo Nakata, Naoyuki Kanayama, Masahiro Morimoto, Isao Miyashiro, Koji Konishi, Risk of thoracic soft tissue sarcoma after breast cancer radiotherapy: a population-based cohort study in Osaka, Japan, *Journal of Radiation Research*, 2024, rrae010, https://doi.org/10.1093/jrr/rrae010

In the Supplementary data file of the originally published version of this article, in Supplementary Table 3, the text “years^3^” was inadvertently omitted from the row entitled “Age at the end of follow-up”.

This has been corrected.

