# Supplementary material for: Risk of thoracic soft tissue sarcoma after breast cancer radiotherapy: a population-based cohort study in Osaka, Japan
Source: J Radiat Res. 2024 Mar 21;65(3):272–8. doi: 10.1093/jrr/rrae010 (PMC11115440; doi:10.1093/jrr/rrae010)

**Supplementary Table 1.** Patient characteristics.

|  | Radiotherapy | |  |
| --- | --- | --- | --- |
| Characteristic | Yes, N = 13,762^1^ | No, N = 27,658^1^ | P-value^2^ |
| Age at breast cancer diagnosis, years |  |  | < 0.001 |
| 20–29 | 124 (0.9%) | 165 (0.6%) |  |
| 30–39 | 1,169 (8.5%) | 1,746 (6.3%) |  |
| 40–49 | 3,954 (29%) | 6,529 (24%) |  |
| 50–59 | 3,956 (29%) | 7,149 (26%) |  |
| 60–69 | 3,137 (23%) | 6,516 (24%) |  |
| 70–79 | 1,275 (9.3%) | 4,344 (16%) |  |
| 80–84 | 147 (1.1%) | 1,209 (4.4%) |  |
| Median age at breast cancer diagnosis, years | 53.0 (46.0, 62.0) | 57.0 (48.0, 67.0) | < 0.001 |
| Age at the end of follow-up, years |  |  | < 0.001 |
| 21–29 | 9 (< 0.1%) | 11 (< 0.1%) |  |
| 30–39 | 141 (1.0%) | 218 (0.8%) |  |
| 40–49 | 821 (6.0%) | 1,283 (4.6%) |  |
| 50–59 | 2,681 (19%) | 4,049 (15%) |  |
| 60–69 | 3,882 (28%) | 6,430 (23%) |  |
| 70–79 | 3,998 (29%) | 7,950 (29%) |  |
| 80–100 | 2,230 (16%) | 7,717 (28%) |  |
| Median age at the end of follow-up, years | 68.0 (59.0, 76.0) | 71.0 (62.0, 81.0) | < 0.001 |
| Chemotherapy |  |  | < 0.001 |
| No | 7,509 (55%) | 15,294 (55%) |  |
| Yes | 5,949 (43%) | 12,219 (44%) |  |
| Unknown | 304 (2.2%) | 145 (0.5%) |  |
| Hormone therapy |  |  | < 0.001 |
| No | 4,664 (34%) | 14,436 (52%) |  |
| Yes | 8,635 (63%) | 12,705 (46%) |  |
| Unknown | 463 (3.4%) | 517 (1.9%) |  |
| Progressive stage |  |  | < 0.001 |
| Carcinoma in situ | 923 (6.7%) | 1,561 (5.6%) |  |
| Localized | 8,706 (63%) | 16,138 (58%) |  |
| Regional lymph node metastasis | 3,803 (28%) | 8,770 (32%) |  |
| Adjacent organ invasion | 330 (2.4%) | 1,189 (4.3%) |  |
| ^1^n (%); median (interquartile range) | | | |
| ^2^Pearson’s Chi-squared test; Wilcoxon rank sum test | | | |

**Supplementary Table 2.** Multivariable Poisson regression analysis of the relative risk of thoracic angiosarcoma

| Characteristic | Person-years | Event N | RR^1^ | 95% CI^1^ | P-value |
| --- | --- | --- | --- | --- | --- |
| Radiotherapy |  |  |  |  |  |
| No | 361,946 | 3 | — | — |  |
| Yes | 175,768 | 9 | 6.70 | 1.94, 30.8 | 0.005 |
| Age at the end of follow-up, years^2^ |  |  |  |  |  |
| 21–69 | 216,816 | 3 | — | — |  |
| 70–100 | 320,899 | 9 | 2.85 | 0.84, 13.0 | 0.12 |
| Calendar year of breast cancer diagnosis |  |  |  |  |  |
| 1990–1999 | 248,595 | 3 | — | — |  |
| 2000–2010 | 289,119 | 9 | 2.30 | 0.67, 10.6 | 0.2 |
| Chemotherapy |  |  |  |  |  |
| No/unknown | 297,142 | 8 | — | — |  |
| Yes | 240,573 | 4 | 0.76 | 0.19, 2.66 | 0.7 |
| Hormone therapy |  |  |  |  |  |
| No/unknown | 246,012 | 6 | — | — |  |
| Yes | 291,702 | 6 | 0.70 | 0.22, 2.29 | 0.5 |
| Progressive stage |  |  |  |  |  |
| Early | 374,678 | 9 | — | — |  |
| Advanced | 163,036 | 3 | 1.03 | 0.21, 3.86 | > 0.9 |
| ^1^RR = relative risk, CI = confidence interval  ^2^Two categories (21–59 years and 60–69 years) were merged. | | | | | |

**Supplementary Table 3.** Multivariable Poisson regression analysis of the relative risk of other subtypes of thoracic soft tissue sarcoma

| Characteristic^1^ | Person-years | Event N | RR^2^ | 95% CI^2^ | P-value |
| --- | --- | --- | --- | --- | --- |
| Radiotherapy |  |  |  |  |  |
| No | 361,946 | 1 | — | — |  |
| Yes | 175,768 | 6 | 10.4 | 1.76, 198 | 0.031 |
| Age at the end of follow-up, years^3^ |  |  |  |  |  |
| 21–59 | 83,714 | 4 | — | — |  |
| 60–100 | 454,000 | 3 | 0.17 | 0.03, 0.79 | 0.022 |
| Chemotherapy |  |  |  |  |  |
| No/unknown | 297,142 | 5 | — | — |  |
| Yes | 240,573 | 2 | 0.45 | 0.06, 2.48 | 0.4 |
| Progressive stage |  |  |  |  |  |
| Early | 374,678 | 5 | — | — |  |
| Advanced | 163,036 | 2 | 1.38 | 0.17, 7.57 | 0.7 |
| ^1^Covariates of calendar year of breast cancer diagnosis and hormone therapy were excluded.  ^2^RR = relative risk, CI = confidence interval  ^3^Two categories (60–69 years and 70–100 years) were merged. | | | | | |

**Supplementary Table 4.** Multivariable Poisson regression analysis including age at breast cancer diagnosis instead of age at the end of follow-up

| Characteristic | Person-years | Event N | RR^1^ | 95% CI^1^ | P-value |
| --- | --- | --- | --- | --- | --- |
| Radiotherapy |  |  |  |  |  |
| No | 361,946 | 4 | — | — |  |
| Yes | 175,768 | 15 | 7.26 | 2.56, 26.0 | < 0.001 |
| Age at breast cancer diagnosis, years |  |  |  |  |  |
| 20–49 | 199,041 | 5 | — | — |  |
| 50–59 | 148,924 | 5 | 1.31 | 0.36, 4.71 | 0.7 |
| 60–84 | 189,749 | 9 | 1.94 | 0.66, 6.39 | 0.2 |
| Calendar year of breast cancer diagnosis |  |  |  |  |  |
| 1990–1999 | 248,595 | 4 | — | — |  |
| 2000–2010 | 289,119 | 15 | 2.32 | 0.81, 8.31 | 0.15 |
| Chemotherapy |  |  |  |  |  |
| No/unknown | 297,142 | 13 | — | — |  |
| Yes | 240,573 | 6 | 0.68 | 0.22, 1.91 | 0.5 |
| Hormone therapy |  |  |  |  |  |
| No/unknown | 246,012 | 7 | — | — |  |
| Yes | 291,702 | 12 | 1.20 | 0.48, 3.28 | 0.7 |
| Progressive stage |  |  |  |  |  |
| Early | 374,678 | 14 | — | — |  |
| Advanced | 163,036 | 5 | 1.18 | 0.35, 3.42 | 0.8 |
| ^1^RR = relative risk, CI = confidence interval | | | | | |

**Supplementary Fig. 1.** Cumulative incidence of thoracic angiosarcoma.


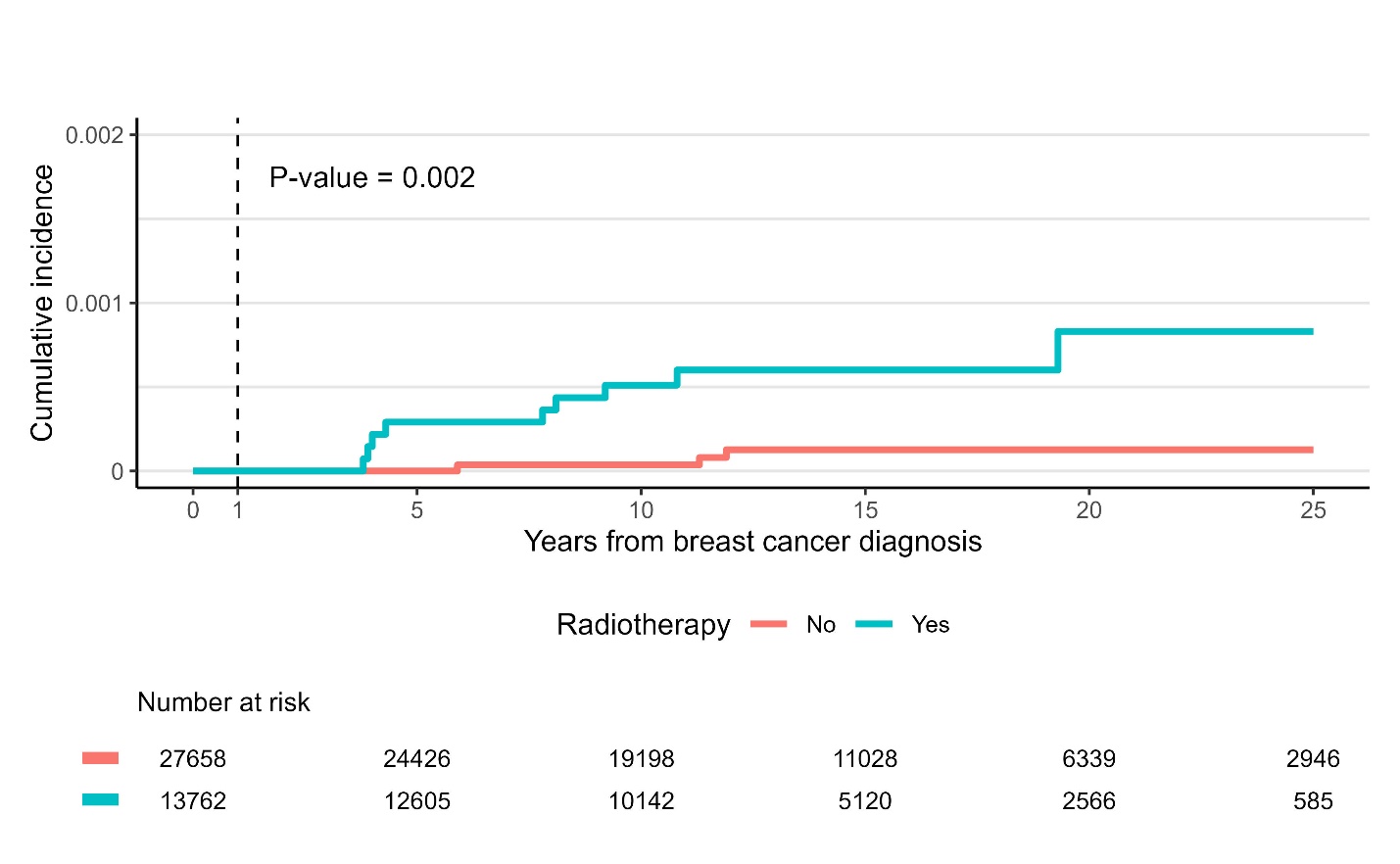


**Supplementary Fig. 2.** Cumulative incidence of other subtypes of thoracic soft tissue sarcoma.
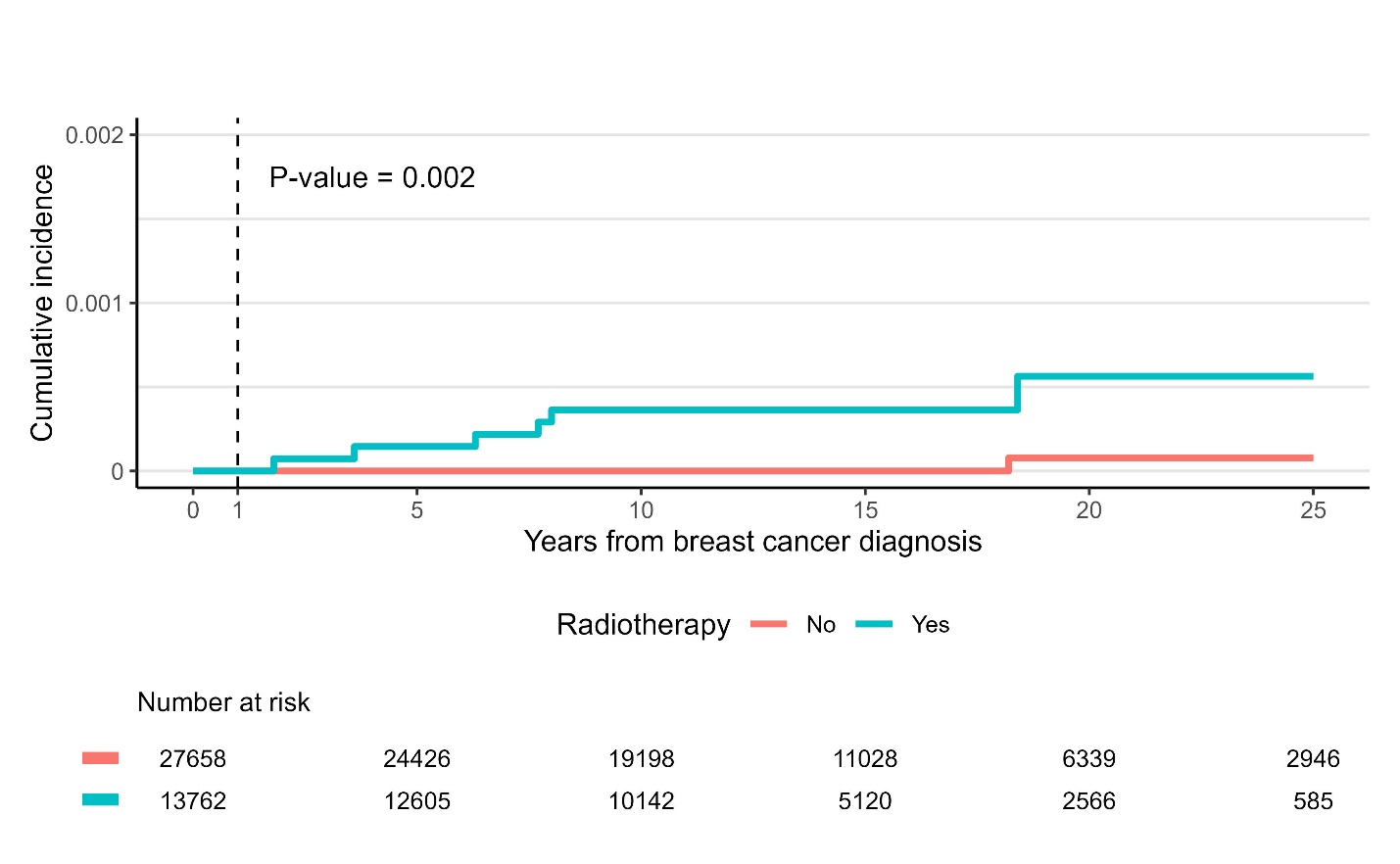

Supplement: Supplemental_data_rrae010 [file supplemental_data_rrae010.docx]
